# Nisin and Nisin Probiotic Disrupt Oral Pathogenic Biofilms and Restore Their Microbiome Composition towards Healthy Control Levels in a Peri-Implantitis Setting

**DOI:** 10.3390/microorganisms10071336

**Published:** 2022-07-01

**Authors:** Allan Radaic, Hanna Brody, Fernando Contreras, Maryam Hajfathalian, Luke Lucido, Pachiyappan Kamarajan, Yvonne L. Kapila

**Affiliations:** 1 Department of Orofacial Sciences, School of Dentistry, University of California San Francisco, San Francisco, CA 94143, USA; allan.radaic@ucsf.edu (A.R.); hanna.brody@ucsf.edu (H.B.); fernando.contreras@ucsf.edu (F.C.); m.hajfathalian@gmail.com (M.H.); luke.lucido@ucsf.edu (L.L.); pachiyappan.kamarajan@ucsf.edu (P.K.); 2 Department of Radiology, University of Pennsylvania, Philadelphia, PA 19104, USA; 3 Division of Oral and Systemic Health Sciences, Sections of Biosystems and Function and Periodontics, School of Dentistry, University of California, Los Angeles, Los Angeles, CA 90095, USA

**Keywords:** nisin, *Lactococcus lactis*, oral biofilm, peri-implantitis, titanium discs, dental implants

## Abstract

Peri-implantitis is characterized by chronic inflammation of the peri-implant supporting tissues that progressively and irreversibly leads to bone loss and, consequently, implant loss. Similar to periodontal disease, oral dysbiosis is thought to be a driver of peri-implantitis. However, managing peri-implantitis with traditional treatment methods, such as nonsurgical debridement or surgery, is not always successful. Thus, novel strategies have been proposed to address these shortcomings. One strategy is the use of probiotics as antimicrobial agents since they are considered safe for humans and the environment. Specifically, the probiotic *Lactococcus lactis* produces nisin, which has been used worldwide for food preservation. The objective of this study was to determine whether nisin and the wild-type (WT) nisin-producing *L. lactis* probiotic can disrupt oral pathogenic biofilms and promote a healthier oral microbiome within these oral biofilms on titanium discs. Using confocal imaging and 16S rRNA sequencing, this study revealed that nisin and WT *L. lactis* probiotic disrupt oral pathogenic biofilms in a peri-implantitis setting in vitro. More specifically, nisin decreased the viability of the pathogen-spiked biofilms dose-dependently from 62.53 ± 3.69% to 54.26 ± 3.35% and 44.88 ± 2.98%, respectively. Similarly, 10^5^ CFU/mL of WT *L. lactis* significantly decreased biofilm viability to 52.45 ± 3.41%. Further, both treatments shift the composition, relative abundance, and diversity levels of these biofilms towards healthy control levels. A total of 1 µg/mL of nisin and 10^3^ CFU/mL of WT *L. lactis* were able to revert the pathogen-mediated changes in the Proteobacteria (from 80.5 ± 2.9% to 75.6 ± 2.0%, 78.0 ± 2.8%, and 75.1 ± 5.3%, respectively) and Firmicutes (from 11.6 ± 1.6% to 15.4 ± 1.3%, 13.8 ± 1.8%, and 13.7 ± 2.6%, respectively) phyla back towards control levels. Thus, nisin and its nisin-producing *L. lactis* probiotic may be useful in treating peri-implantitis by promoting healthier oral biofilms, which may be useful for improving patient oral health.

## 1. Introduction

Since the beginning of humankind, humans have tried to replace their missing teeth. It is well documented that the ancient Egyptians, in 2500BCE, stabilized periodontally missing teeth using ligatures made of golden wires [1]. However, the introduction of the two-stage osseointegrated, titanium, root-form dental implant in the late 1960s revolutionized dentistry [1,2].

**Figure 1:**
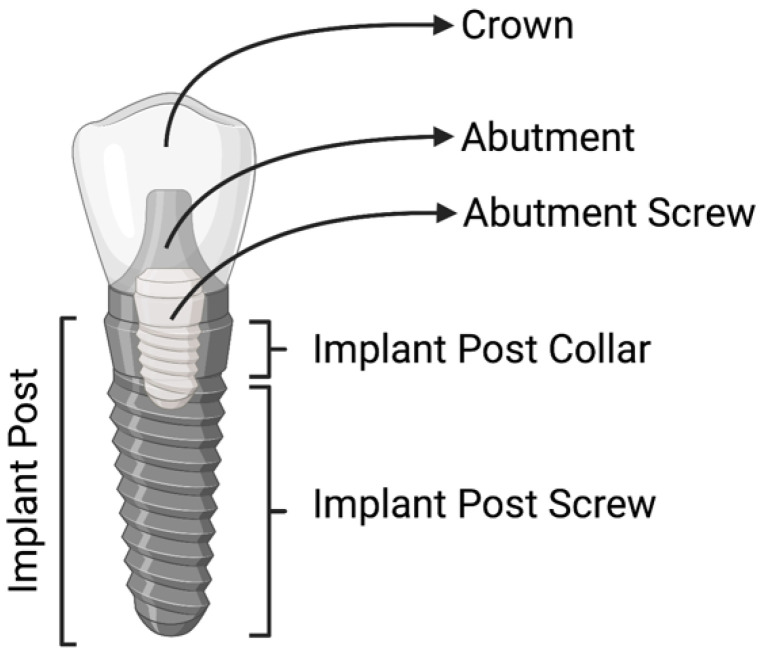
Dental implant structure.

Modern dental implants consist of four main parts (Figure 1)—the crown, abutment, abutment screw, and implant post, which can be further divided into the collar and the screw [3]. The crown is usually fabricated from metal and ceramic [3]. The abutment and the implant post are made of titanium alloy, typically grade V, which is a titanium alloy strengthened by the inclusion of 6% Aluminum and 4% Vanadium (also known as Ti-6Al-4V). This alloy promotes optimal osseointegration and can be texturized to improve its properties. Among these textures, the Laser-Lok^®^ surface, in particular, is made of optimally sized microchannels on the implant post collar and screw to help attach and organize both osteoblasts and fibroblasts while eliciting inhibition of epithelial downgrowth and increased attachment of bone and connective tissues to the implant [4,5,6,7].

These structural properties have led to modern dental implants becoming very successful, and recent studies indicate an eight-fold increase in implant prevalence in the partially edentulous US population in 2015–2016 compared to 1999–2000, with an average covariate-adjusted increase in dental implant prevalence of 14% per year, achieving a market share of USD 4.3 billion dollars in 2021 [8,9]. Within the US population, implant prevalence is estimated to reach 23% by 2026 and a revenue forecast of USD 8 billion by 2028 [8,9]. Drivers of this trend include, but are not limited to, the increased size of the geriatric population and the prevalence of tooth-related diseases.

Among these tooth-related diseases, peri-implant diseases are an emerging area of concern, as partial edentulism is not limited to missing natural teeth but also loss of dental implants. Further, peri-implant mucositis and peri-implantitis remain an issue for dental implant morbidity and loss. For instance, a recent review reported the prevalence of peri-implant diseases and found that 23.9–88.0% of patients and 9.7–81.0% of implants presented peri-implant mucositis, while 8.9–45.0% of patients and 4.8–23.0% of implants presented peri-implantitis [10]. Mirroring periodontal disease progression [11], peri-implant mucositis is the first stage of peri-implant disease, characterized by inflammation of the peri-implant soft tissues without bone loss; whereas peri-implantitis is the later, more chronic phase, characterized by chronic inflammation of the peri-implant mucosa that progressively and irreversibly leads to bone loss, decreased osseointegration and consequently, implant loss [2,12,13,14].

The oral microbiome is a complex ecosystem comprised of a thousand microbial species that thrive in a very dynamic environment—the oral cavity—and establishes several host–microbiome interactions with its human host, known as the oralome [11,15]. Despite its resilience, the oralome can succumb to perturbations that cause it to shift as a result of an unbalanced state, known as oral microbiome dysbiosis. This dysbiosis has been increasingly related to local oral diseases, such as caries, periodontitis and peri-implantitis, as well as systemic diseases, such as cardiovascular diseases, including atherosclerosis, Alzheimer’s disease, diabetes, and pregnancy complications, such as pre-term birth [11,16]. Moreover, oral dysbiosis has also been associated with head and neck cancer, especially oral squamous cell carcinoma, as well as gastrointestinal, lung, prostate, breast and uterine cancers [15,17].

Frequently, a spectrum of bacteria is detected in peri-implant lesions and recent data have indicated that peri-implantitis dysbiosis is somehow similar to periodontitis shifts [14,18]. A systematic review on peri-implantitis found similar microbial pathogens in peri-implantitis and periodontitis lesions, including *Tannarella forsythia*, *Porphyromonas gingivalis*, several *Fusobacterium* species, including *Fusobacterium nucleatum*, *Prevotella intermedia*, *Prevotella nigrescens* and *Aggregatibacter actinomycetemcomitans* [19]. Others report that the microbial composition of peri-implantitis is more complex and that there is a distinct microbiological profile from periodontitis, which includes higher *Treponema denticola*, *Staphylococci*, *Peptostreptococci*, *Streptococci*, enterics, and several yeasts species [19,20,21,22].

In this context, the aim of treatment for peri-implant disease is disease resolution, with no further loss of support [23]. There are currently three main treatment modalities for peri-implantitis—nonsurgical debridement, resective treatment with or without implantoplasty, and reconstructive treatment [24]. However, great variability has been reported in outcomes for all treatment options, which is primarily attributed to patient factors, defect morphology, and the reconstructive methods used. Thus, there is currently no major consensus on the best treatment modality for peri-implant disease [25]. Additionally, the use of local or systemic antibiotics may also induce further dysbiosis in oral and gut microbiomes, and both microbiotas may not be able to recover [26,27]. Therefore, addressing the etiology of peri-implant disease is paramount to the successful resolution of this situation, also further highlighting the urgent need for new treatment strategies.

One of these new strategies is the use of bacteriocins and probiotics to assist in mitigating this dysbiosis by suppressing oral pathogens within the oralome [11,28,29,30]. Recently, the potential for using a nisin bacteriocin and nisin probiotic in biomedical applications has been highlighted [11,15,29,30,31]. Nisin is a class I lantibiotic bacteriocin produced by the Gram-positive bacteria *Lactococcus lactis* [11,30], and it is active against both Gram-positive and Gram-negative bacteria, including *Streptococcus aureus*, *Listeria monocytogenes*, *Fusobacterium nucleatum*, *Porphyromonas gingivalis* and *Treponema denticola* [30,32]. Nisin itself and nisin-producing *Lactococcus lactis* spp. have been used successfully to abrogate infections associated with drug-resistant pathogens, gastrointestinal infections, respiratory tract infections, skin and soft tissue infections, mastitis, head and neck cancer (HNC), and other oral diseases using in vitro and in vivo models [11,15,28,29,31,33]. In addition, studies led by our group demonstrate that nisin and its probiotic (i.e., Wild-type (WT) nisin-producing *Lactococcus lactis*) dose-dependently abrogate the growth of pathogens associated with periodontal disease in their planktonic state, and dose-dependently shift pathogenic oral biofilms (grown on tissue culture plates) towards health, without inducing cytotoxicity to human oral cells [28,32]. Despite these advances, there have been no studies testing nisin and its probiotic in a peri-implantitis setting in the literature so far.

Therefore, the objective of this study was to determine the extent to which nisin, and its probiotic form (nisin-producing *Lactococcus lactis*) can disrupt peri-implantitis-associated pathogenic oral biofilms and drive these pathogenic biofilms back towards health.

## 2. Materials and Methods

### 2.1. Human Saliva Collection and Informed Consent

Human saliva collection was approved by the University of California San Francisco Institutional Review Board (IRB #17-21912, Reference #186994, approved on 25 April 2017). The collection protocol was previously published by our group [28,32]. Using sample size calculation analyses, we estimated that a minimum of 10 patients were required to obtain a power of 0.8 for this study (>0.8 required). In our study, ten healthy volunteers with no known health issues verbally consented to donating saliva. No information from the volunteers was collected at any time prior to or at the time of saliva donation. Prior to the collection, the volunteers were informed not to eat, drink and/or smoke for at least 30 min before the donation. They were comfortably seated and given a sterile tube for saliva collection. About 5–15 mL of saliva was obtained from each volunteer in about 10 min each time. After collection, all samples were immediately kept on ice until further processing. All the collected saliva was pooled, centrifugated (10,000 RPM for 30 min) and separated into a Cell-Containing Saliva (CCS) and Cell-Free Saliva (CFS). The CCS was used as the biofilm inoculum, which was prepared by adding glycerol (50% *v*/*v*) to the precipitate from the centrifuged pooled saliva and stored at −80 °C. CFS was used as biofilm nutrient media, and it was obtained by collecting the supernatant of the centrifuged pooled saliva, diluted with sterile Phosphate Buffer Saline (PBS) (1:4 *v*/*v*) and stored at −80 °C.

### 2.2. Bacteria and Biofilm Growth

Human saliva-derived oral biofilms were grown by adding 20 µL of CCS to 600 µL of CFS per well in 24-well plates containing one 13 mm diameter by 1 mm thick grade V (Ti-6Al-4V) titanium disc with Laser-Lok^®^ surface texture (Biohorizons, Birmingham, AL, USA). The plates were then incubated under aerobic conditions for 48 h at 37 °C in a humidified Thermo-Fisher Forma Series II Incubator (Waltham, MA, USA). CFS media was changed every 24 h. For the pathogen-spiked biofilms, 24 h preformed biofilms were spiked with 6 × 10^5^ CFU/mL of each periodontal pathogen (i.e., *Treponema denticola*, *Fusobacterium nucleatum*, and *Porphyromonas gingivalis*) and incubated under aerobic conditions for another 24 h.

*Treponema denticola* (ATCC 35405), *Porphyromonas gingivalis* (ATCC 33277), and *Fusobacterium nucleatum* (ATCC 25586) were grown as described previously [34,35,36]. *T. denticola* was cultured in Oral Treponeme Enrichment Broth (OTEB; Anaerobe Systems, Morgan Hill, CA, USA), while *P. gingivalis* and *F. nucleatum* were cultured in Brain–Heart Infusion broth (BHI; Sigma-Aldrich, Burlington, MA, USA) supplemented with hemin (5 µg/mL) and vitamin K (1 µg/mL) under anaerobic conditions. Anaerobic conditions were obtained by placing bacterial samples into sealed anaerobic jars that underwent five cycles of depressurization (vacuum formation) and Nitrogen (N_2_) pressurization (1 ATM) and kept at 37 °C in a Fisher-Scientific Isotemp Incubator. The bacteria were split every 3–7 days. To ensure log-phase growth, bacteria were passaged at least once before use in experiments. Previous literature determined that a 0.1 absorbance at 600 nm of *Treponema denticola* [34], *Porphyromonas gingivalis* [37,38], or *Fusobacterium nucleatum* [39] suspension contains 2.4 × 10^8^ CFU/mL, 2.4 × 10^8^ CFU/mL, and 1 × 10^8^ CFU/mL of each of the bacteria, respectively. Purity of the cultures was confirmed by 16S sequencing prior to their use in the experiments.

Nisin-producing *Lactococcus lactis* (ATCC 11454) and non-nisin-producing (NN) *L. lactis* (gift from Cork Institute of Technology, Cork, Ireland) strains were grown in BHI at 37 °C with shaking and under aerobic conditions in an Eppendorf G24 Environmental Incubator Shaker and passaged every 2–3 days. Bacteria were passaged at least once before use in the experiments. Before treatment, the cells were centrifuged (3000 RPM for 5 min) and diluted in PBS for CFU/mL quantification. Previous literature determined that a 0.1 absorbance at 600 nm of *L. lactis* suspension contains 6 × 10^7^ CFU/mL of the bacterium [40]. Then, the cells were further diluted in CFS to obtain the desired CFU/mL concentration for treatment.

### 2.3. Nisin Preparation

Nisin Z^®^P powder (Handary, Brussels, Belgium), which has >95% purity, was gently mixed in Mili-Q water to a concentration of 5 mg/mL in a 15 mL tube covered in aluminum foil. The tube was then placed on a rotator and mixed for 4 h to completely solubilize nisin. Next, the solution was filtered using a 0.22 µm syringe filter. Finally, filtered nisin was further diluted in CFS to the desired concentration for treatment.

### 2.4. Oral Biofilm Disruption

The preformed oral biofilms were challenged with either WT or NN *L. lactis* strains (6 × 10^3^ and 6 × 10^5^ CFU/mL) or nisin (1 or 10 µg/mL) for 24 h. Biofilms were then fixed for 5 min using paraformaldehyde (4% in PBS). Next, the samples were stained for 15 min using LIVE/DEAD BacLight Bacterial Viability Kit (ThermoFisher Scientific, Waltham, MA, USA). This kit uses two dies—Syto9, which is membrane permeable, thus staining all bacterial cells, and Propidium Iodide, which is membrane impermeable, thus staining only cells with damaged cell membranes. Propidium Iodide has a higher DNA affinity, thus displacing Syto9, in cases when both dyes are competing for the site [28]. Then, the samples were washed three times with PBS to remove the excess stain and mounted on slides by adding a small drop of mounting media (Agilent, Santa Clara, CA, USA) on top of the Titanium discs, and a 13 mm coverslip was added on top of the disc to seal the biofilms. Finally, the fluorescence in the biofilm samples was evaluated using a TCS SP8 X Confocal Microscope (Leica, Wetzlar, Germany).

### 2.5. 16S rRNA Sequencing

For microbiome sequencing and analyses, DNA was extracted from all the biofilm samples using a QIAmp DNA Mini Kit (Qiagen, Germantown, MD, USA), according to the manufacturer’s protocol for bacterial samples. Next, the extracted DNA samples were evaluated for purity and concentration using a Nanodrop UV-Vis spectrophotometer (Thermo-Scientific, Waltham, MA, USA) and submitted to Novogene (Sacramento, CA, USA) for 16S rRNA sequencing, according to the company’s 16S rRNA sequencing pipeline. Briefly, the samples underwent a qPCR reaction for amplification of the V4 sequences, using the 515F (5′-GTGCCAGCMGCCGCGGTAA-3′) and 806R (5′-GGACTACHVGGGTWTCTAAT-3′) primers.

After 16S rRNA library generation, the library quality was assessed on the Qubit@ 2.0 Fluorometer (Thermo Scientific) and Agilent Bioanalyzer 2100 system. Then, the libraries were sequenced on Illumina HiSeq platform, and 250 bp paired-end reads were generated at Novogene (Sacramento, CA, USA).

Finally, the raw tags were filtered and developed into clean tags according to QIIME (Boulder, CO, USA—Version 1.7.0) quality-controlled process [41]. After the quality control, clean tags were aligned to the Gold database (Release 20110519), and the chimera sequence was detected by using UCHIME Algorithm (Sonoma, CA, USA—Version 7.0.1001) [42,43]; these non-chimera clean tags were defined as effective tags. The effective tags were clustered into OTUs with ≥ 97% similarity by Uparse (Version 7.0.1001) [44]. The representative sequence for each OTU was selected and the taxonomic information was annotated using the RDP classifier and GreenGene database [45,46]. The library was then analyzed for OTU clusters and phylogenies relationship and species annotation; alpha and beta diversity; unweighted UniFrac and Principal Coordinate Analysis (PCoA) of the Unweighted UniFrac distances.

### 2.6. Statistical Analysis

Data from the oral biofilm disruption and biofilm richness, diversity and unweighted UniFrac indices were analyzed using a one-way ANOVA, and intergroup differences were analyzed by Tukey’s post hoc test; p significance values are displayed in the figure legends. Phyla and Genus OTU data were analyzed by a two-way ANOVA, and intergroup differences were analyzed by Tukey’s post hoc test; *p* significance values are displayed in the figure legends. Oral biofilm disruption data are presented as means ± SD and were derived from duplicate experiments with triplicates for each sample. 16S sequencing data were derived from duplicate experiments with duplicates for each sample. Phyla and genus OTU results are presented as means ± SD, while richness, diversity and unweighted UniFrac indexes are presented as boxplots (medians, minimums and maximums).

## 3. Results

### 3.1. Nisin and Nisin-Producing Probiotic Disrupt Oral Biofilms on Titanium Discs

**Figure 2:**
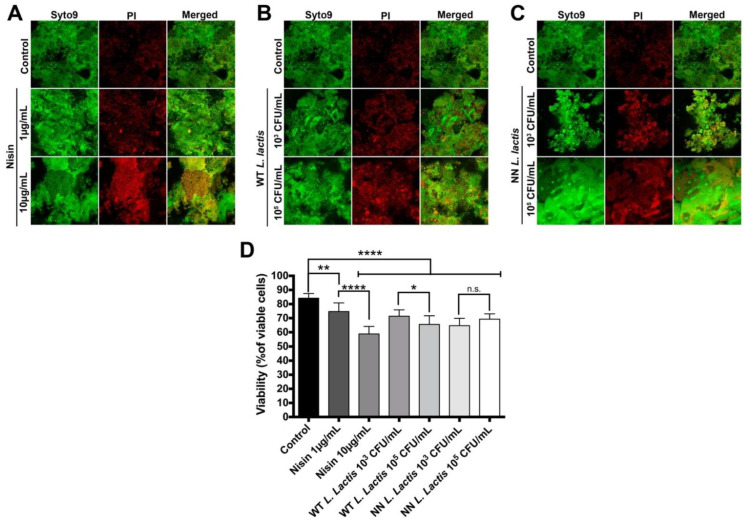
Nisin effectively disrupts oral biofilms on Laser-Lok^®^ grade V titanium discs. Biofilms were treated with either nisin (**A**), WT *L. lactis* (**B**) or NN *L. lactis* (**C**) for 24 h. (**A**–**C**) Representative images of fluorescently labeled biofilms are shown. The columns, from left to right, represent the different staining dyes/protocols; SYTO9 is a live cell stain, whereas propidium iodide (PI) is a dead-cell stain, and the merged image shows the overlap of both stains. The rows represent the different treatments. (**D**) Quantification of the bacterial viability assessed from the confocal images is shown. n.s. means not significant; * means *p* ≤ 0.05; ** means *p* ≤ 0.01; and **** means *p* ≤ 0.0001 between the marked samples.

The ability of nisin (Figure 2A), wild-type nisin-producing *Lactococcus lactis* (WT *L. lactis*, Figure 2B) and a non-nisin-producing *Lactococcus lactis* control (NN *L. lactis*, Figure 2C) to disrupt 48 h preformed human oral saliva-derived biofilms grown on titanium discs was examined, and changes in biofilm viability were quantified (Figure 2D). Control biofilms exhibited 84.15 ± 3.35% viability. Nisin significantly decreased biofilm viability dose-dependently to 74.72 ± 6.10% and 58.80 ± 5.31%, respectively. Similarly, WT *L. lactis* significantly decreased biofilm viability dose-dependently to 71.37 ± 4.32% and 65.55 ± 6.18%, respectively. Interestingly, both concentrations of NN *L. lactis* significantly decreased biofilm viability to 64.64 ± 5.25% and 69.29 ± 3.77% compared to control, although not dose-dependently.

### 3.2. Nisin and Nisin-Producing Probiotic Disrupt Pathogen-Spiked Oral Biofilms on Titanium Discs

**Figure 3:**
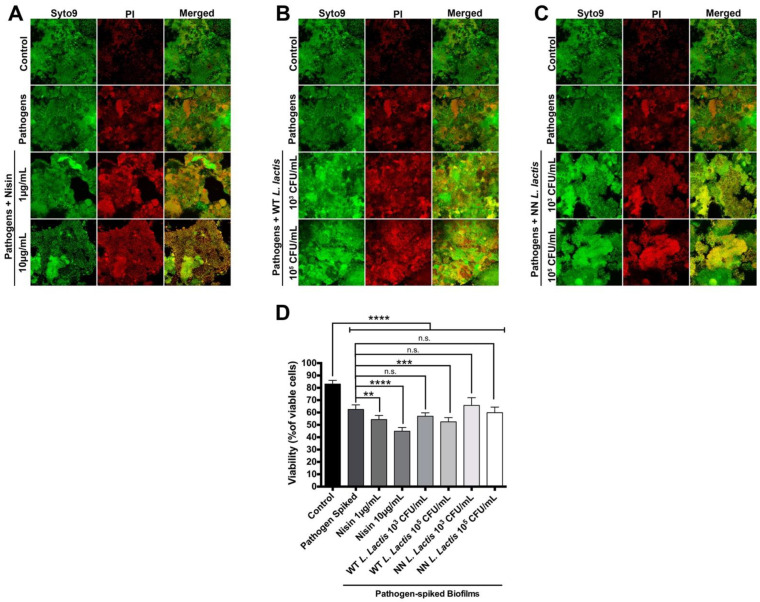
Nisin effectively disrupts pathogen-spiked oral biofilms on Laser-Lok^®^ grade V titanium discs. Pathogen-spiked biofilms were treated with either nisin (**A**), WT *L. lactis* (**B**) or NN *L. lactis* (**C**) for 24 h. (**A**–**C**) Representative images of fluorescently labeled biofilms are shown. The columns, from left to right, represent the different staining dyes/protocols; SYTO9 is a live cell stain, propidium iodide (PI) is a dead-cell stain, and the merged images show the overlap of both stains. The rows represent the different treatments. (**D**) Quantification of bacterial viability assessed from the confocal images is shown. n.s. means not significant; ** means *p* ≤ 0.01; *** means *p* ≤ 0.001; and **** means *p* ≤ 0.0001 between the marked samples.

Next, oral biofilms were spiked with known peri-implantitis pathogens, namely *T. denticola*, *F. nucleatum* and *P. gingivalis*, and the ability of nisin (Figure 3A), WT *L. lactis* (Figure 3B) and NN *L. lactis* (Figure 3C) to abrogate these pathogen-spiked biofilms was evaluated, and changes in biofilm viability were quantified (Figure 3D).

Control biofilms again exhibited a viability of 83.04 ± 3.00%. Interestingly, spiking the biofilms with the pathogens significantly decreased biofilm viability to 62.53 ± 3.69%. Nisin further decreased the viability of the pathogen-spiked biofilms dose-dependently to 54.26 ± 3.35% and 44.88 ± 2.98%. Similarly, 10^5^ CFU/mL of WT *L. lactis* significantly decreased biofilm viability to 52.45 ± 3.41%. On the other hand, 10^3^ CFU/mL of WT *L. lactis* and both concentrations of NN *L. lactis* did not significantly decrease the pathogen-spiked biofilm viability.

### 3.3. Microbiome Sequencing of Titanium-Derived Oral Biofilms Reveals Unique and Divergent Species upon Spiking with Pathogens

**Figure 4:**
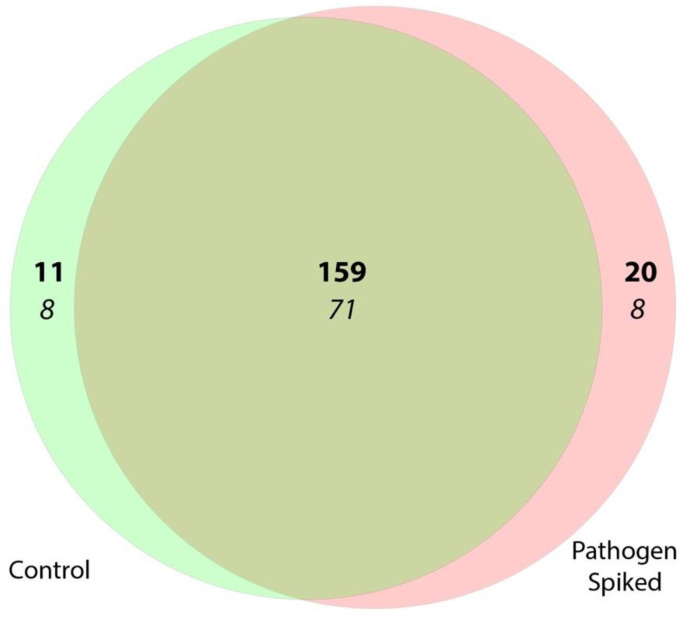
Distinct species and genera found in the pathogen-spiked biofilms compared to control biofilms. Venn diagram showing the comparisons of the microbiome of the different biofilms with the number of genera (**bold**) and species (*italic*) found in the control biofilms (green) and in the pathogen-spiked biofilms (red).

16S rRNA sequencing of control oral biofilms grown on titanium discs and those spiked with pathogens revealed that both biofilms shared 159 genera and 71 species and diverged in 31 genera and 16 species—11 genera and 8 identified species were unique to the healthy control biofilm and 20 genera, and 8 identified species were unique to the pathogen-spiked biofilm (Figure 4). A list containing the divergent genera and species can be found in Table 1.

### 3.4. Nisin and WT L. lactis Probiotic Shift Specific Phyla and Genera in Pathogen-Spiked Oral Biofilms Back towards Control Levels

**Figure 5:**
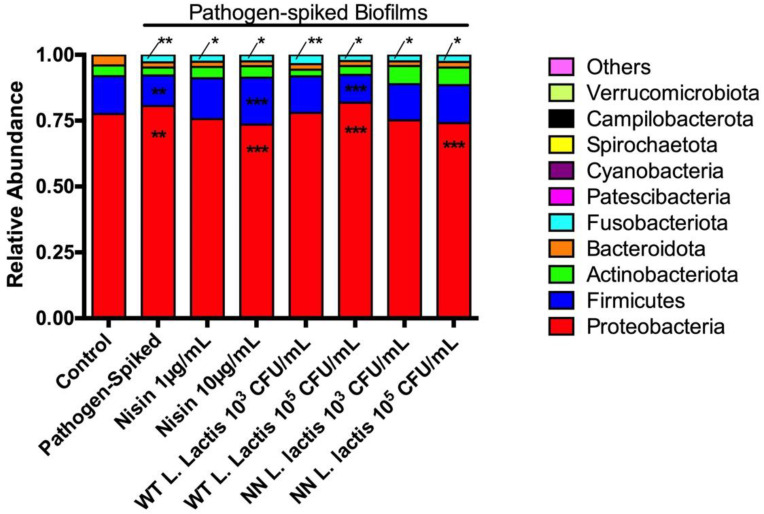
Lower doses of nisin and probiotic revert Proteobacteria and Firmicutes phyla back to control levels. Top 10 phyla relative abundance are displayed; * means *p* ≤ 0.05; ** means *p* ≤ 0.01, *** means *p* ≤ 0.001 between the marked sample and control.

Next, we evaluated Phyla relative abundance in the samples (Figure 5). The healthy control biofilms revealed a high preponderance of the Proteobacteria phylum (77.5 ± 3.4%), followed by Firmicutes (14.4 ± 1.5%), Actinobacteriota (4.1 ± 0.7) and Bacteroidota (3.9 ± 1.6%) phyla. Compared to healthy control biofilm, the pathogen-spiked biofilms showed a significant increase in Proteobacteria (to 80.5 ± 2.9%) and Fusobacteriota phyla levels (from 0.7 ± 0.6% to 2.75 ± 0.9%), followed by a significant decrease in the Firmicutes phylum levels (to 11.6 ± 1.6%). Although the Bacteroidota phylum seemed to be decreased in the pathogen-spiked biofilms compared to the healthy controls, the decrease was not statistically significant. Importantly, 1 µg/mL of nisin, and 10^3^ CFU/mL of WT and NN *L. lactis* were able to revert the pathogen-mediated changes in the Proteobacteria (to 75.6 ± 2.0%, 78.0 ± 2.8%, and 75.1 ± 5.3% respectively) and Firmicutes (to 15.4 ± 1.3%, 13.8 ± 1.8%, and 13.7 ± 2.6%, respectively) phyla back towards control levels. Additionally, except for WT *L. lactis* 10^3^ CFU/mL, all treatments were able to significantly decrease the elevated Fusobacteria phylum levels mediated by pathogen-spiking of the biofilms (from *p* = 0.0062 (pathogen-spiked) to a range from *p* = 0.0130 (Nisin 1 µg/mL) to *p* = 0.0469 (WT *L. lactis* 10^5^ CFU/mL)), although these levels were still significantly different than the healthy control.

Interestingly, 10 µg/mL of nisin significantly decreased the Proteobacteria phylum (to 73.5 ± 1.3%), compared to the control but significantly increased the Firmicutes relative abundance (to 17.8 ± 0.7%), compared to the control. A similar significant effect was mediated by 10^5^ CFU/mL of NN *L. lactis* on the Proteobacteria phylum (further decreased to 74.1 ± 2.8%, compared to control). Intriguingly, 10^5^ CFU/mL of WT *L. lactis* did not affect the pathogen-spiked Proteobacteria/Firmicute pattern. Other changes in the remaining top 10 phyla were not statistically significant, compared to the healthy and pathogen-spiked controls.

**Figure 6:**
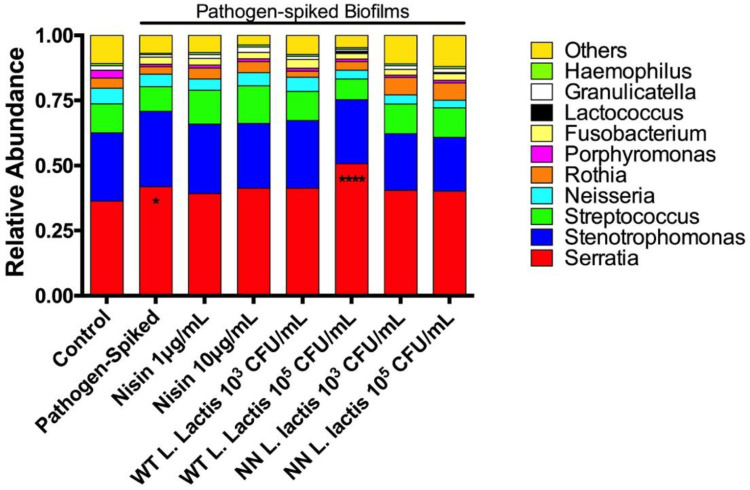
Lower doses of nisin and probiotic revert the *Serratia* genus back to control levels. Top 10 genera relative abundance are displayed; * means *p* ≤ 0.05; while **** means *p* ≤ 0.0001 between the marked sample and control.

Next, we evaluated Genera relative abundance in the samples (Figure 6). The healthy control biofilms were co-dominated by *Serratia* (36.3 ± 5.1%) and *Stenotrophomonas* (26.2 ± 3.1%) genera, followed by *Streptococcus* (11.1 ± 0.8%), *Neisseria* (6.0 ± 1.3%), *Rothia* (3.9 ± 0.7%), and *Porphyromonas* (3.0 ± 1.5%). Compared to the healthy biofilms, the pathogen-spiked biofilms showed a significant increase in the *Serratia* levels (to 41.9 ± 7.2) as the dominant member in the biofilms. Except for WT *L. lactis* 10^5^ CFU/mL, all treatments were able to significantly decrease *Serratia* levels back to healthy control levels. Similar to findings in the Phyla relative abundance, WT *L. lactis* 10^5^ CFU/mL further increased *Serratia* levels (to 50.7 ± 9.1%). Other changes in the remaining top 10 genera were not statistically significant compared to the healthy and pathogen-spiked controls.

### 3.5. Nisin and WT L. lactis Probiotic Shift Alpha and Beta Diversity Indices, as Well as the Principal Coordinate Analysis (PCoA) Distances in Pathogen-Spiked Oral Biofilms Back towards Control Levels

**Figure 7:**
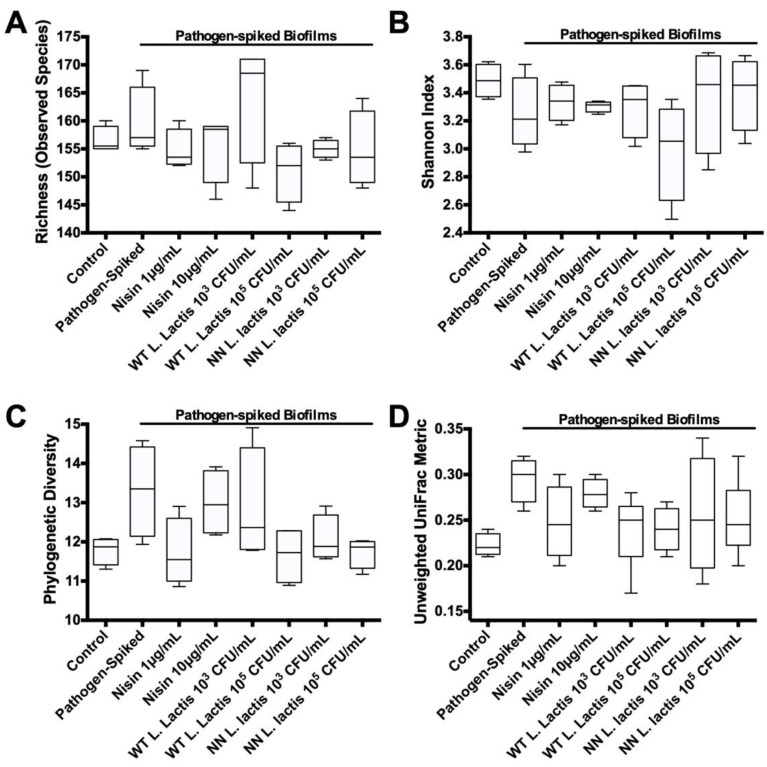
Nisin and probiotic show a tendency to revert pathogen-spiked biofilm richness and diversity indices back to control levels. (**A**) Species richness (number of observed species); (**B**) Shannon diversity index; (**C**) phylogenic diversity index; (**D**) unweighted UniFrac metric.

Next, we analyzed the alpha and beta diversity of the biofilms. For these analyses, we evaluated the species richness (Figure 7A), Shannon diversity (Figure 7B), phylogenic diversity (Figure 7C) and UniFrac index (Figure 7D).

Species richness refers to the number of species within a defined area or sample [47]. The healthy control biofilms revealed a median of 155.5 (155.0–160.0) species, whereas the pathogen-spiked biofilms exhibited a slight increase to 157.0 (155.0–169.0) species. Although, this difference was not statistically significant. Except for WT *L. lactis* 10^3^ CFU/mL, all treatments mediated small decreases in the median species richness with a tendency toward shifting the numbers back to control levels. Interestingly, treatment with WT *L. lactis* 10^3^ CFU/mL led to a median of 168.5 (148.0–171.0) species. Nonetheless, these changes were not statistically significant.

The Shannon diversity index takes into account not only species richness but also the proportion of each species in an ecosystem, thus giving a better description of the ecosystem’s diversity [48]. The healthy control biofilms exhibited a Shannon diversity index of 3.487 (3.354–3.622), whereas the pathogen-spiked biofilms showed a slight diversity index decrease to 3.212 (2.977–3.603). Except for WT *L. lactis* 10^5^ CFU/mL, all treatments resulted in a slight tendency toward increasing the Shannon diversity index back to control levels. Interestingly, WT *L. lactis* 10^5^ CFU/mL treatment led to a diversity index of 3.055 (2.497–3.353). Although, none of these changes was statistically significant.

The phylogenetic diversity (PD) index takes a different approach to describing diversity, as it delineates the total amount of phylogenetic distance among species within a community [49]. Phylogenetic distance measures provide far more power than abundance measures, as they exploit the degree of divergence between different sequences [50]. The healthy control biofilms exhibited a PD Index of 11.87 (11.30–12.08), whereas the pathogen-spiked biofilms revealed a slight increase to 13.35 (11.94–14.48). All the treatments show a decreased trend in the median PD index compared to the pathogen-spiked biofilms, although none of these changes were statistically significant.

The unique fraction metric (UniFrac) measures the phylogenetic distance in the phylogenetic tree as the fraction of the branch length of the tree between two samples or regions. In this sense, UniFrac is a Beta-diversity index. There are two versions of UniFrac—The unweighted UniFrac metric considers only species presence and absence and counts the fraction of branch length unique to either community, whereas weighted UniFrac metric weighs the branch length with species abundance information. The Unweighted UniFrac index is most efficient in detecting abundance change in rare lineages [50]. The healthy control biofilms exhibited a UniFrac metric of 0.22 (0.21–0.24), whereas the pathogen-spiked biofilms revealed a slight increase to 0.30 (0.26–0.32). All treatments were able to slightly decrease this index back to control levels, although none of these changes was statistically significant.

**Figure 8:**
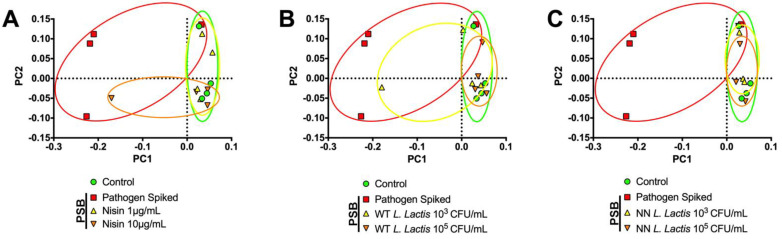
Nisin and the probiotic effectively bring PCoA variables/distance of the pathogen-spiked oral biofilms back toward control levels. Images show the Principal Coordinate Analysis (PCoA) of the unweighted UniFrac distances for control biofilms and pathogen-spiked biofilms (PSB) treated with (**A**) Nisin 1 and 10 µg/mL, (**B**) WT *L. lactis* 10^3^ and 10^5^ CFU/mL and (**C**) NN *L. lactis* 10^3^ and 10^5^ CFU/mL. Colored ellipses highlight sample group distance.

Finally, we analyzed the unweighted Unifrac distances via Principal Coordinate Analysis (PCoA) for nisin (Figure 8A), WT *L. lactis* (Figure 8B) and NN *L. lactis* (Figure 8C). PCoA transforms the original UniFrac metrics into a new set of orthogonal axes [51]. In the resulting plot, samples coordinated closer to one another are more similar than those ordinated further away. Even though the pathogen-spiked biofilms had some similarities with the healthy control biofilms, its PCoA area and distance were more spread out than the healthy control area. Nisin 1 µg/mL, WT *L. lactis* 10^5^ CFU/mL and both NN *L. lactis* concentrations were able to significantly reduce the pathogen-spiked area and shift the area/ellipse back toward the healthy control area. Interestingly, Nisin 10 µg/mL and WT *L. lactis* 10^3^ CFU/mL were able to partially reduce the area/spread, but not completely.

## 4. Discussion

Oral biofilms play an essential role both in the development of natural oral physiology and defense of the host [11,52]. Because of these important roles, an imbalance in the oral microbiome or dysbiosis within these oral biofilms is associated with a variety of oral and systemic diseases, including Alzheimer’s disease, head and neck cancer, periodontal disease, caries, and peri-implantitis [11,15,18]. Although antibiotics can be used to address this dysbiosis, recent data indicate that antibiotics may even induce dysbiosis in such a way that the oral microbiota cannot recover [26,27]. Peri-implant diseases are in part biofilm mediated, and exacerbation of disease can lead to loss of implants over time. Thus, new strategies to modulate oral biofilm dysbiosis, especially those associated with peri-implantitis, are needed.

Previously, our group has demonstrated that a nisin-producing WT *L. lactis* inhibits and disrupts in vitro human-derived oral biofilms and shifts the bacterial population within these biofilms towards health [28,32]. Additionally, the probiotic has been considered safe and well-tolerated by head and neck patients receiving chemotherapy [53]. However, we were unable to find any studies testing the probiotic or nisin in peri-implantitis settings. Therefore, the objective of the current study was to test whether nisin and its probiotic (WT *L. lactis*) can disrupt pathogenic peri-implantitis biofilms and shift their dysbiotic composition back to a healthier state in vitro.

Overall, our results indicate that both nisin and WT *L. lactis* can reduce the viability of pathogen-spiked biofilms while also reverting the pathogen-mediated biofilm changes, including significantly restoring Proteobacteria and Firmicutes phyla relative abundance toward healthy control levels, significantly restoring the *Serratia* genus back toward healthy control levels, and driving richness and diversity indices and metrics back toward healthy control levels. Thus, based on these results, the use of 1 µg/mL of nisin or 6 × 10^3^ CFU/mL of WT *L. lactis* for 24 h may improve oral health in a peri-implantitis setting.

Lin et al. [54] tested plantaricin 149 (Pln149) produced by *Lactobacillus plantarum* NRIC 149 on *P. gingivalis* and *Prevotella intermedia* biofilms grown on Ti discs in vitro. The authors demonstrated that 100 µg/mL of Pln149 given for 24 h significantly decreased the integrated optical density (IOD) of the biofilms. Additionally, the authors showed that 125 µg/mL of Pln149 presented no significant cytotoxicity to bone marrow stromal cells. In our study, we demonstrated that 1 µg/mL of nisin can significantly reduce the viability of the human saliva-derived oral biofilms grown on Ti discs, while a previous report from our group [32] demonstrated no nisin cytotoxicity on gingival fibroblasts, periodontal ligament cells, oral keratinocytes and osteoblast-like cells up to 400 µg/mL.

On the probiotic side, *Lactobacillus reuteri* is one of the most studied probiotics for potential peri-implantitis treatment [22]. The probiotic has antagonistic effects on peri-implantitis pathogens [22], yet, recent metadata analyses demonstrated that the bacterium provides limited benefits to patients with peri-implant mucositis or peri-implantitis [55,56]. For instance, Peña et al. [57] tested the use of *L. reuteri* after mechanical debridement and the use of a0.12% chlorhexidine mouthwash in 50 patients with peri-implant mucositis. The placebo control showed that mechanical debridement and chlorhexidine mouthwash did improve all clinical variables, except for probing pocket depth, whereas the probiotic did not provide any additional clinical benefit.

Martorano-Fernandes et al. [58] tested the efficacy of *Streptococccus salivarius* on *Candida albicans* biofilms grown in vitro on titanium surfaces under reduced oxygen levels. The authors found that *S. salivarius* did not hinder *C. albicans* virulence, suggesting the probiotic would be ineffective in an in vivo peri-implantitis setting.

Interestingly, our results demonstrate WT *L. lactis* may be effective in an in vivo peri-implantitis setting. WT *L. lactis* probiotic mediates a dual action on biofilms—it produces nisin, which by itself can abrogate oral biofilms [28,32], and the bacterium itself may compete for nutrients and space inside the biofilms [28]. Therefore, we included a control *L. lactis* strain, which does not produce nisin (i.e., non-nisin producing *L. lactis*; NN *L. lactis*) in our experimental approach. This probiotic control would affect the biofilms only through nutritional and space competition, thereby removing the overall effects of nisin with the treatment. Even though NN *L. lactis* was not able to significantly decrease the viability of pathogen-spiked biofilms, such as the wild-type nisin-producing probiotic, it was still able to drive significant changes in the pathogen-spiked biofilm relative abundance. These data point out that the viability effect may be solely or in great part due to nisin, whereas probiotic competition may play a greater role in driving relative abundance changes to the pathogen-spiked biofilms. Interestingly, a similar effect was found in our previous study on periodontitis, where, again, NN *L. lactis* was not able to significantly decrease the viability of the biofilms while still driving changes in the relative abundance and diversity indexes [28]. Likewise, our group demonstrated similar NN *L. lactis* nutritional effects in an in vivo polymicrobial infection model recently [59]. NN *L. lactis* were able to significantly decrease the level of some pathogens (i.e., *P. gingivalis* and *F. nucleatum*) compared to the infection control, indicating that the probiotic does affect these, at least partially and potentially, via nutrient and space competition. However, WT *L. lactis* was still able to reduce significantly more *F. nucleatum* and *T forsythia* compared to NN *L. lactis*, indicating that nisin adds an additional antibacterial effect over these periopathogens. Surprisingly, nisin and WT *L. lactis* significantly reduced alveolar bone loss, and the oral and systemic inflammatory host response, while significantly enhancing the population of fibroblasts and osteoblasts at the infection sites. In contrast, NN *L. lactis* did not significantly promote any of these effects, indicating that they are mediated via nisin. All this underscores how important it is to evaluate bacterial composition and competition in in vitro and in vivo microbiome settings, especially when working with bacteriocin-producing probiotics.

The use of 16S rRNA sequencing can be seen as a potential limitation in this study. Even though the oral microbiome has a predominance of bacterial species, it is also comprised of other kingdoms, such as fungi, viruses, and protozoa species [11,15]. Furthermore, interkingdom interactions are known to take place within the oralome [11]. For instance, Janus et al. [60] demonstrated that *C. albicans* alters the bacterial microbiome in early in vitro oral biofilms, resulting in the increased abundance of anaerobic bacteria, such as *Veillonella*, *Prevotella*, *Leptotrichia*, and *Fusobacterium* genera under oxygen-rich conditions. Gao et al. [61] found that a polybacterial infection in mice promoted a shift in the virome, establishing an increased association of Gammaretrovirus genera, Golden hamster intracisternal A-particle H18, Bat gammaretrovirus and Porcine type-C oncovirus species with an increase in bone loss in vivo. 16S rRNA sequencing uses highly conserved regions of the bacterial and archeon genome (i.e., the gene coding for the ribosomal small (16S) unit RNA) to identify these microorganisms [62], thus limiting the range of taxons analyzed by the technique. This can be mitigated by the use of metagenome shotgun sequencing, which does not have any taxonomic limitations. However, this technique still remains much more expensive, both experimentally and computationally, than 16S rRNA sequencing [62].

Another limitation of this study is the in vitro nature of the experimentation. Even though saliva from healthy patients was collected, the oral biofilms were grown in vitro on titanium discs, which cannot replicate the oral host–microbiome interactions (oralome), especially the interaction of the microbiome with epithelial cells and the host immune response [11]. An in vivo model, such as a polymicrobial infection mouse model [59,61], could be used to address this limitation.

Finally, to the best of our knowledge, this is the first time that nisin and WT *L. lactis* have been tested in a peri-implantitis setting, as we were unable to find similar studies testing nisin and the probiotic in this setting.

## 5. Conclusions

In conclusion, nisin and WT *L. lactis* were able to significantly disrupt peri-implantitis pathogenic oral biofilms, returning their relative abundance, composition, and diversity indices back toward healthy control levels. Based on these results, the use of 1 µg/mL of nisin or 10^3^ CFU/mL of WT *L. lactis* for 24 h may improve oral health in a peri-implantitis setting.

## Figures and Tables

**Table 1:** Distinct genera and species found between control and pathogen-spiked biofilms.

Control Biofilms		Pathogen-Spiked Biofilms	
Genera	Species	Genera	Species
Selenomonas	*Veillonellaceae bacterium*	Bifidobacterium	*Bifidobacterium dentium*
Oribacterium	*Oribacterium* sp.	Selenomonas	*Schwartzia* sp.
Filifactor	*Filifactor alocis*	Veillonella	*Veillonella* sp.
Rikenellaceae RC9 gut group	*Bacteroidales oral*	Shuttleworthia	*Shuttleworthia satelles*
Chryseobacterium	*Chryseobacterium soldanellicola*	Massilia	*Massilia timonae*
Slackia	*Slackia exigua*	Gracilibacteria	*Gracilibacteria bacterium*
*Prevotella*	*Prevotella loescheii*	*Prevotella*	*Prevotella saccharolytica*
Treponema	*Treponema socranskii*	Treponema	*Treponema lecithinolyticum*
Pelomonas	*-*	*Fusobacterium*	*-*
Gemella	*-*	Phenylobacterium	*-*
Comamonas	*-*	Olsenella	*-*
		Reyranella	*-*
		Pseudarcicella	*-*
		Mycoplasma	*-*
		Family XIII UCG-001	*-*
		Bacteroides	*-*
		Fretibacterium	*-*
		Rheinheimera	*-*
		Chloroplast	*-*
		Campylobacter	*-*

## Data Availability

The data presented in this study are available upon request to the corresponding author.
